# Validation of the Grobman model for successful vaginal birth after cesarean section in Jordanian women

**DOI:** 10.1186/s12884-023-05385-z

**Published:** 2023-01-20

**Authors:** Hasan Rawashdeh, Haneen Aljalodi, Rafeef Abu shamleh, Sumyah Alshorman, Heba AboShindi, Shatha Awawdeh

**Affiliations:** 1 Obstetrics and Gynecology Department, Jordan University of Science and Technology, Amman, Jordan; 2 Obstetrics and Gynecology Department, King Abdulla University Hospital, Ar-Ramtha, Jordan; 3 USAID Health Services Quality Accelerator Activity / University Research Co., Amman, Jordan; 4 King Abdullah II School for Information Technology, The University of Jordan, Amman, Jordan

**Keywords:** Vaginal Birth after Cesarean Section (VBAC), Cesarean section repeat, Validation, Jordan

## Abstract

**Background:**

To validate both models of Grobman nomogram (The antenatal and the intrapartum model) for predicting successful intended Vaginal Birth After Caesarean delivery (VBAC) in a Jordanian population.

**Methods:**

A retrospective study has identified all live, singleton, term, cephalic pregnancies with a previous lower segment cesarean section who opted for a Trial Of Labour After Caesarean Section (TOLAC) between January 2014 to December 2020. Five variables were used for the antenatal model, while ten variables were used for the intrapartum model. Two sets of patients were created: one for the antenatal model and the other for the intrapartum model. The predicted probability for each woman was calculated and compared with the successful VBAC for each category. The predictive ability was assessed with a receiver operating characteristic, and the area under the curve (AUC) was determined.

**Results:**

There were seven hundred and fourteen complete cases for the antenatal model and six hundred ninety-seven for the intrapartum model. Our population's overall number of VBAC is 83.89% for the antenatal group and 82.92% for the intrapartum group. The mean predicted probability for a successful intended VBAC using the antenatal and intrapartum models were 79.53 ± 13.47 and 78.64 ± 14.03, respectively. The antenatal and intrapartum predictive models ROC had an AUC of 65% (95% CI: 60%-71%) and 64% (95% CI: 58%-69%), respectively.

**Conclusions:**

Both models are validated in the Jordanian population. Adapting the antenatal model as supporting evidence can lead to a higher rate of TOLAC.

## Background

Despite the general consensus released by the WHO in 1985, which stated that the cesarean section rate should not exceed 15% for any region, [1] the rate of cesarean section continued to rise steadily worldwide [2–4]. This exceedingly escalating rate of cesarean section has created a group of women with an obstetric history of a previous cesarean section who will face a complex discussion concerning the mode of delivery after their first cesarean section scar [5].

In Jordan, the situation is complicated for two reasons. First, evidence suggests that the cesarean section rate reached 30 percent for all deliveries in 2012 [6]. Second, the average lifetime births for a woman in Jordan is about 3.7 [7]. Thus, a good percentage of women will be placed in this situation where they must decide the mode of delivery after a previous cesarean section scar.After one lower segment cesarean section, women can choose to deliver by cesarean section electively (known as Elective Repeat Caesarean Section, ERCS), or they may try to deliver vaginally (Known as Trial of Labour After Caesarean Section, TOLAC). If they have opted vaginal birth trial, they may end up with either a successful vaginal birth (Known as Successful TOLAC or Vaginal Birth After Caesarean Section, VBAC), or they may end up with an unsuccessful TOLAC (known as Emergency Caesarean Section, ECS).

TOLAC is considered a safe mode of delivery for most women with a previous lower segment cesarean section carrying singleton fetuses, suggested by the Royal College of Obstetricians and Gynaecologists RCOG and the American College of Obstetricians and Gynaecologists [8, 9]. Women should be aware that maternal mortality rate is rare in both options.

Successful TOLAC or VBAC carries the best maternal outcome. In contrast, unsuccessful TOLAC or ECS carries the worst maternal outcome. It is associated with higher maternal morbidity and mortality rates [10]. The probability of having a successful VBAC is a cornerstone element in the discussion session. Thus, an accurate prediction of a successful VBAC can lead to more refined counseling, lower the decisional conflict, and improve the VBAC attempt rates [11].

In general, the success rate of VBAC is about 72–75%. This rate differs between individuals according to many factors.8 These factors may present antenatally, like maternal age, maternal BMI, the indication of the previous cesarean section, and the presence of vaginal delivery and a successful VBAC. In comparison, other factors are identified only intrapartum such as fetal weight, head station, and cervix condition by the time of labor. Although the above factors help guide women toward the best delivery option, they are not specific for each woman, and hence they cannot provide a unique risk versus success rate assessment.

Several researchers looked for factors that affect the success rate of VBAC. Some have looked for factors that are negatively associated with VBAC, and they have developed a scoring system based on points accumulation like Troyer and Parisi. [12] Other researchers developed a nomogram to provide a continuous probability prediction for a given outcome, like Grobman [13].

The first and the second nomogram models described by Grobman during the antenatal and intrapartum periods are the most popular models. Both models were developed prospectively to predict a personalized probability [13, 14]. They were also validated using different populations in the USA, Europe, and Japan [15–17]. However, Grobman models were not validated to be used in Jordan. Hence, this paper aims to validate the antenatal and intrapartum models by Grobman on a Jordanian population.

## Methods

This study is a retrospective study conducted at a major teaching hospital in the north of Jordan. Data were collected from the medical registry department after receiving the institutional review board approval number (2019/658). All women with a previous one lower segment cesarean section who were admitted to the labor ward for delivery between January 2014 to December 2020 were reviewed.

The inclusion criterion included all term pregnancies with a previous lower segment cesarean section carrying live cephalic fetuses admitted for a trial of vaginal birth. The exclusion criteria were multiple pregnancies, extended scars or scars other than transverse lower uterine segment, and preterm deliveries.

Collected data was categorized as follow:

1. Demographic data: Maternal age, maternal height, maternal weight at booking and during the last antenatal visit, hypertensive disorders in pregnancy.
2. Obstetrics data: Gestational age at delivery, number of previous vaginal deliveries, number of previous vaginal birth after cesarean section, and the indication of the previous cesarean section, whether it was due to failure to progress as a potentially recurrent cause or not, and the presence of hypertensive disorder.
3. Intrapartum data: Whether labor was induced or not, cervical dilatation and effacement on admission, and station of the fetal head on admission.
4. The primary outcome was either VBAC or emergency cesarean section.

After identifying cases that have met the inclusion criteria, we have examined the completeness of data entries required for Grobman's antenatal and intrapartum model. We ended up with two sets of cases: one having completed data for the antenatal model, the second for the intrapartum model.

In order to validate Grobman's antenatal model, we have used the five variables described in the antenatal equation (excluding the racial background). The variables are maternal age, BMI at booking, history of vaginal delivery before cesarean section, history of vaginal birth after cesarean section, and a recurring indication of the cesarean section.

The predictive probability for the antenatal group was calculated using the following equation = exp (w)/ [1 + exp(w)], where w = 3.766–0.039 (age) –0.060 (pre-pregnancy body mass index) + 0.888 (any prior vaginal delivery) + 1.003 (vaginal delivery after prior caesarean) – 0.632 (recurring indication for caesarean) [13].

In order to validate Grobman's intrapartum model, we have used ten out of the eleven variables described in the intrapartum equation (excluding the racial background). The variables are maternal age, BMI at the last antenatal visit, history of vaginal delivery before cesarean section, history of vaginal birth after cesarean section, a recurring indication of the cesarean section, hypertensive disorders, cervical effacement (in percent) and cervical dilatation (in centimeters) on admission, fetal head station (from -5 to + 3), and labor induction.

The predictive probability for the intrapartum group was calculated using the following equation = exp (w) /[1 + exp(w)], where w = 7.059 − 0.037(age) − 0.044 (BMI) + 0.955 (any prior vaginal delivery) + 0.851 (vaginal delivery after prior cesarean) − 0.655 (recurring indication for cesarean) − 0.109 (estimated gestational age at delivery) − 0.499 (hypertensive disease of pregnancy) + 0.044 (effacement) + 0.109 (dilation) + 0.082 (station) − 0.452 (labor induction) [14].

Regarding the ethnic background, most of our patients descended from the same background. Also, this variable was omitted from the currently used model; therefore, it was not used during the calculation.

### Preliminary analysis

Statistical analysis was conducted using Statistical Package for Social Sciences (SPSS) version 23 [18]. Double-checking for the data entry was performed to prevent data entry errors. After that, tendency measures, minimum, maximum, and frequency measures were conducted to screen data for any outliers or missing data. For continuous variables, such as BMI, boxplots and histograms showed positive skewness distribution for some variables. Accordingly, a non-parametric test was conducted for research purposes [19].

### Normality

The mean score of the main study variables was computed. Normality was evaluated using frequency distribution, skewness, and kurtosis values near zero between -1 to + 1 [19].

## Results

There were seventeen thousand two hundred seventy-one deliveries between January 2014 and December 2020. Of them, five thousand four hundred and one patients had a singleton, term cephalic pregnancy with a previous cesarean delivery. Only one thousand one hundred and thirty-seven opted for TOLAC (21%), while four thousand two hundred and sixty-four decided to have an elective repeat cesarean section. Of the one thousand one hundred and thirty-seven patients who have met the criteria for inclusion, seven hundred and fourteen cases have complete data for the antenatal model. Six hundred and ninety-seven have complete data for the intrapartum model. Our population's overall number of VBAC is 83.89% for the antenatal group and 82.92% for the intrapartum group.

### Participant characteristics

The mean age of women in the antenatal group was 31.96 years (SD = 4.72), ranging from 20 to 45. In comparison, the mean age for the intrapartum group was 32 years (SD = 4.73), ranging from 20 to 45 years. The mean score of BMI for the antenatal group was 29.36 (SD = 3.64), with a range of 19 – 42. In comparison, the mean score of BMI among the intrapartum group was 29.36 (SD = 4.63), ranging from 19.5- 42.

Ninety-six percent of both groups had no previous hypertensive disorders. Most women in the intrapartum model had more than 3 cm dilated cervix (88.5%). About half of the recruited intrapartum women had effacement of 45%, and 87% were not induced for delivery.

### Factors influencing VBAC among the antenatal group

As shown in Table 1, no statistical differences in the mean rank of age and BMI that reflectVBAC or ERCS. In contrast, the presence of hypertensive disorders during pregnancy was statistically significant in affecting the mode of delivery between VBAC and ERCS. This means that pregnant women with no hypertensive disorders were more likely to have VBAC than others. strongest association was between the previous history of vaginal birth and a successful VBAC χ2(2, 714) = 21.35; *p* < 0.001). For example, non-hypertensive women with a previous history of vaginal birth were more likely to have a successful VBAC.

**Table 1:** Factors influencing vaginal birth after caesarean section among the antenatal group

Variable	VBAC	ERCS	Statistical test used	*P*-value
Age			U = 31,780.5	**0.188**
** n**	599	115		
** Mean rank**	361.94	334.35		
BMI			U = 30,805	**0.07**
** n**	599	115		
** Mean rank**	351.43	389.13		
Presence of HTN disorders			*X*^*2*^ = 11.14^a^	**0.004**
** No HTN**	581 (97%)	107 (93%)		
** Preeclampsia**	17 (2.8%)	5 (4.3%)		
** Chronic HTN**	1 (0.2%)	3 (2.6%)		
History of vaginal birth			*X*^*2*^ = 21.35^b^	**0.0001**
** No history of VB**	162 (27.0%)	56 (48.7%)		
** History of VB before CS**	131 (21.9%)	17 (14.8%)		
** History of VBAC**	306 (51.1%)	42 (36.5%)		

*X*^*2*^ Chi Square test, *U* Mann–Whitney test, *HTN* Hypertension, *VB* Vaginal Birth, *VBAC* Vaginal Birth After Caesarean section, *CS* Caesarean Section

^a^ < 0.05

^b^ < 0.001

### Factors influencing VBAC among the intrapartum group

As shown in Table 2, no statistical differences in the mean rank of age and BMI reflect having a VBAC or ERCS. A measure of the central tendencies of both groups was tested using the Mann–Whitney U test, which revealed a statistically significant difference between both groups based on the gestational age of the participants (U (697) = 28,666.5, *p* < 0.05).women with a previous history of VBAC were more likely to deliver vaginally than women who had no previous VBAC (χ2 (1, 697) = 5.24; *p* < 0.05).

**Table 2:** Factors influencing vaginal birth after caesarean section among the intrapartum group

Variable	VBAC	ERCS	Statistical test used	*P*-value
Age			t = 0.754	**0.451**
** n**	578	119		
** M (SD)**	32.07 (4.68)	31.71 (5.00)		
BMI			U = 30586.5	**0.057**
** n**	578	119		
** Mean rank**	342.42	380.97		
Gestational Age			U = 28823.5^a^	**0.005**
** n**	578	119		
** Mean**	339.25	395.79		
Previous VB			*X*^*2*^ = 3.93^a^	**0.029**
** Yes**	200 (34.6%)	30 (25.2%)		
Previous VBAC			*X*^*2*^ = 5.24^a^	**0.014**
** Yes**	290 (50.2%)	46 (38.7%)		
Induction			*X*^*2*^ = 7.40^a^	**0.007**
** Yes**	64 (11.1%)	24 (20.2%)		
Station			*X*^*2*^ = 1.135	**0.769**
** -3**	347 (60.0%)	69 (58.0%)		
** -2**	110 (19.0%)	27 (22.7%)		
** -1 and 0**	112 (19.4%)	22 (18.5%)		
** + 1 and + 2**	9 (1.6%)	1 (0.8%)		
Cervical effacement on admission			*X*^*2*^ = 18.642^b^	**0.0001**
** 0–30%**	41 (7.1%)	16 (13.4%)		
** 40–50%**	152 (26.3%)	48 (40.3%)		
** 60–70%**	106 (18.3%)	18 (15.1%)		
** 80% or more**	279 (48.3%)	37 (31.1%)		
Cervical dilation on admission			*X*^*2*^ = 25.164^b^	**0.0001**
** Closed**	2 (0.3%)	1 (0.8%)		
** 1–2**	53 (9.2%)	23 (19.3%)		
** 3–4**	261 (45.2%)	68 (57.1%)		
** 5 or more**	262 (45.4%)	27 (22.7%)		
Presence of HTN disorders			*X*^*2*^ = 6.76^a^	**0.034**
** No HTN**	559 (96.7%)	111 (93.3%)		
** Preeclampsia**	19 (3.3%)	7 (5.9%)		
** Chronic HTN**	0 (0.0%)	1 (0.8%)		

*X*^*2*^ Chi Square test, *U* Mann–Whitney test, *HTN* Hypertension, *VD* Vaginal Birth, *VBAC* Vaginal Birth After Caesarean section, *CS* Caesarean Section

^a^ < 0.05

^b^ < 0.001

The strongest association observed was between a successful VBAC and the advanced cervical effacement and cervical dilatation on admission.

In otherwords, women with a previous history of VBAC and presented to the labour ward in active labour had the greatest chance to have a successful VBAC.*Validation of Grobman`s model.*

In order to validate the prediction model, an individual probability of achieving VBAC was calculated for both data sets (Antenatal and intrapartum group). The predictive probability of having VBAC was determined using the previously published Grobman equation for each group, as explained in the methods section. After getting the prediction probabilities in percentage as continuous data, they were categorized into ten deciles (0–10%, 11–20%, 21–30%, 31–40%, 41–50%, 51–60%, 61–70%, 71–80%, 81–90%-91–100%) in each category. Then, the actual proportion of VBAC was determined in each category.

Both data sets show acceptable calibration, especially in the high-probability ranges. Table 3 shows good calibration for the antenatal model. The H–L statistic showed a *P*-value of 0.9. The actual probability of VBAC for each decile was higher than the expected in all deciles, except in the 91–100% decile. Likely wise, Table 4 shows the predicted and observed probabilities of successful VBAC for the intrapartum women`s group, according to the Grobman model.

**Table 3:** Predicted and observed probabilities of successful vaginal birth among the antenatal group, according to Grobman model

Predicted probability VBAC (%)	Number of pregnant women (n)	Attended VB	Observed Probability	Confidence Interval 95% (Lower – Upper)
0–10	0	0	0	NA
11–20	0	0	0	NA
21–30	0	0	0	NA
31–40	6	3	50.0	41.80- 67.10
41–50	8	5	62.5	59.12- 92.26
51–60	52	36	69.2	63.60- 79.01
61–70	124	96	77.4	70.20 – 82.67
71–80	116	96	82.8	69.35 – 89.91
81–90	186	162	87.1	- 68.1 – 110.7
91–100	222	201	90.5	-67.94- 97.05

*VB* Vaginal Birth, *VBAC* Vaginal Birth After Caesarean section

**Table 4:** Predicted and observed probabilities of successful vaginal birth among the intrapartum group, according to Grobman model

Predicted probability VBAC (%)	Number of pregnant women (n)	Attended VB	Observed Probability	Confidence Interval 95% (Lower – Upper)
0–10	0	0	0	NA
11–20	0	0	0	NA
21–30	1	1	100.0%	NA
31–40	8	6	75.0%	- 50.31 – 87.74
41–50	13	10	76.9%	- 65.54- 91.27
51–60	56	38	67.9%	- 55.88 – 88.81
61–70	118	89	75.4%	53.48 – 90.81
71–80	118	99	83.9%	68.90 – 94.98
81–90	183	156	85.2%	63.46 – 93.96
91–100	200	179	89.5%	67.09 – 109.63

*VB* Vaginal Birth, *VBAC* Vaginal Birth After Caesarean section

**Fig. 1:**
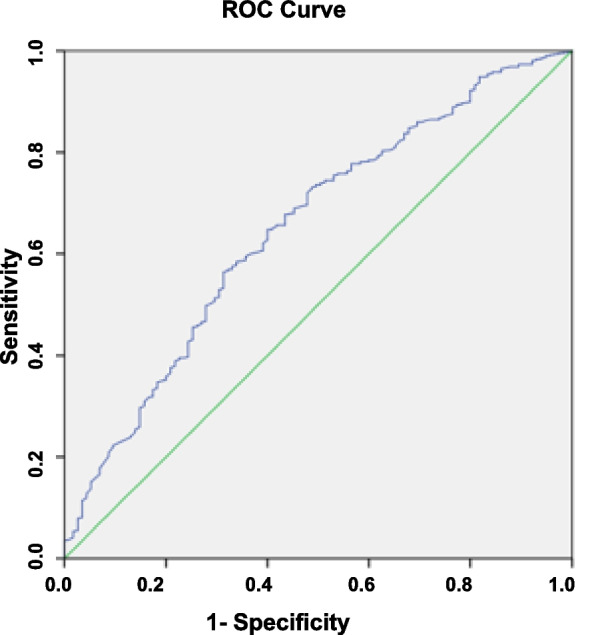
Antenatal model ROC curve

**Fig. 2:**
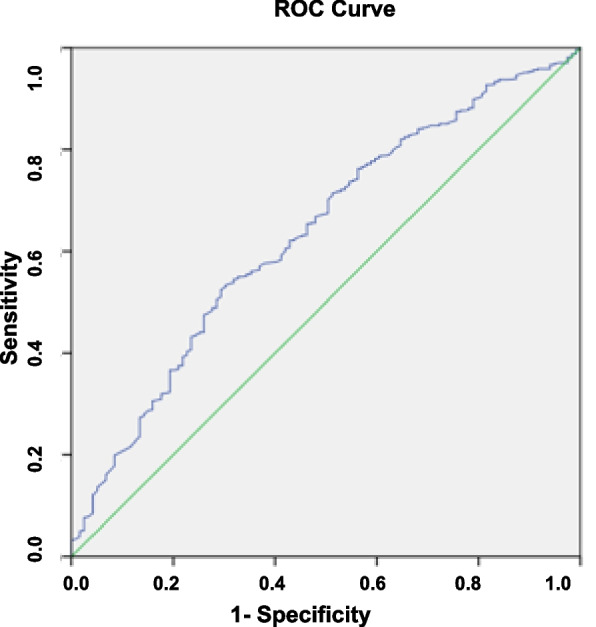
Intrapartum model ROC curve

The Receiver Operating Characteristic (ROC) was conducted to assess the discriminative performance. ROC is considered as a systematic analytical tool for calculating the impact of variability among different threshold of decision. The ROC was obtained by plotting sensitivity against 1- specificity. This predictive model defined the sensitivity as a fraction of the correctly predicted VBAC. Meanwhile, the specificity was reflected by the fraction of emergency caesarean section due to unsuccessful TOLAC that was correctly predicted by the model. Area Under the Curve (AUC) of the ROC was used to assess the prediction model's ability to discriminate between the high and low probability of achieving a VBAC in women, (Note Figs. 1 and 2).

### Discriminant performance

The discriminant performance of the predictive model is shown in Fig. 1 below. The ROC for the antenatal group predictive model had an AUC of 65% (95% CI: 60%-71%). In comparison, the ROC for the intrapartum group predictive model had an AUC of 64% (95% CI: 58%-69%).

### Predictive performance

As shown in Fig. 1, the overall calibration of both predictive models was good. The mean successful VBAC rate intended for the antenatal and intrapartum groups in the current study was 79.5% and 78.6%, respectively. The mean predicted probability for a successful intended VBAC using the antenatal and intrapartum models were 79.53 ± 13.47% and 78.64 ± 14.03%, respectively.

## Discussion

This study is the first to validate Grobman models among the Jordanian population. We have tested both the antenatal and intrapartum models on our population. The main objective of this validation is to act on the high rate of cesarean section by selecting candidates for vaginal birth after cesarean section and supporting their decision. This decision is particularly crucial for women who are planning for large families. These women may end up having more than four cesarean sections knowing that the average number of live births for a Jordanian family is about 3.7. On the other hand, a proper counselling based on a validated model could have saved them from the repeated caesarean sections if they have tried TOLAC rather than an elective repeat cesarean section [7].

Our population's overall number of VBAC is 83.89% for the antenatal group and 82.92% for the intrapartum group. This success rate of VBAC at our institution is considered among the highest numbers worldwide, being very similar to the Australian number and preceded only by the Japanese number, which sets at 91.5% [17, 20].

The mean predicted probability for a successful intended VBAC using the antenatal and intrapartum models were 79.53 ± 13.47% and 78.64 ± 14.03%, respectively. This suggests a good overall calibration for both models as a predictive tool for success. Hence, it can be a handy tool during counselling as an evidenced opinion of success.

The Discriminant performance of the predictive model is generally good, as the ROC of the antenatal model had an AUC of 65% (95% CI: 60%-71%). In comparison, the ROC of the intrapartum model had an AUC of 64% (95% CI: 85%-69%). Compared with other validation studies, the AUC we got is the lowest among all similar studiescarried out on different populations. For example, the AUC was 81% for the Japanese and the Ethiopian populations, 77% for Grobman's population, 71% for the Australian population, 69% for the French population, and 68% for the Italian population [13, 17, 20–23].

Five reasons can explain this difference in the AUC. First: In our study, not all the variables of Grobman's model were significantly associated with the prediction of VBAC in the validation of the model. For example, maternal age and BMI did not contribute to the prediction in both models of our study.

Second: Many other variables in our cohort did not show the expected influence as per Grobman's model, which can explain why additional intrapartum variables did not improve the AUC. Therefore, we do encourage future research groups to study their own demographic variables in addition to the variables described by Grobman to create a modified model that may show a higher AUC.

Third: There is a noticeable difference in the BMI between our cohort and different populations. The Ethiopian and French validation studies previously described the difference in BMI as a possible cause of different results. The BMI in our population was 29.3 for both groups, while it was 21.5, 26.4, 25.8, and 28 for the Japanese, original Grobman, Ethiopian, and French populations [13, 16, 17, 21, 23]. This difference might suggest tha presence of different factors that have been influencing the outcome without being considered.

Fourth: A drop in the AUC was also noticed when the Grobman model was used to validate another group of women from the same population that the original model relied on (USA) [24]. Suggesting that different factors may have been influencing the outcome without being considered.

Fifth: Most of our population opted for an elective repeat cesarean section in the ratio of (4:5). At the same time, our VBAC success rate is relatively high, setting at 83%. These numbers may suggest a problem in the counseling sessions. It may reflect that clinicians were not supporting the option of TOLAC for all mothers equally. In contrast, adequate support for TOLAC was provided only for mothers with a high chance of a successful VBAC. This bias during counseling can be responsible for the skewed data being very crowded in the 91–100% centile. Also, it can explain the low AUC we got because of the small proportions in the remaining centiles. A careful evaluation and monitoring of the TOLAC counseling sessions should be provided, and a personalized antenatal prediction model should be encouraged. In addition, a clear pamphlet released to mothers helps them to discuss the birth mode with their family members or friends before the actual counseling session.

At the end, it is worth to mention that Grobman`s model under predicts successful VBAC in the lower ranges which could potentially dissuade patients from considering TOLAC. Therefore, careful clinical review before considering TOLAC is recommended in these cases. This finding might be attributed to the limited number of participants who met the lower ranges in the study.

## Conclusions

Both nomogram models developed by Grobman appear to be applicable to the Jordanian population. The discriminant performance of both predictive models was generally good. Both models can be adapted as supportive evidence at antenatal clinics, where adequate support and encouragement should be provided when the probability is more than 50% of a VBAC.

Although both models are applicable, the antenatal model is easier and faster, and shows a better outcome as it is using five variables rather than ten and vaginal examination is not necessary. Itcan also be used early in pregnancy,thereby allowing enough time for the mothers to decide on their birth mode.

We would suggest the coming researchers describe new different demographic variables in addition to the variables described by Grobman, aiming at creating a modified model that may have a higher AUC. Lastly, it is also encouraged to study the maternal and neonatal outcomes and incorporate them into the outcome.

## Data Availability

The data that support the findings of this study are available from King Abdulla University Hospital (KAUH). Restrictions apply to the availability of these data, which were used under license for this study. Data are available [Hasan Rawashdeh] with the permission of the IRB committee at KAUH. Collection and management of data was according to the guidelines and regulations provided by the King Abdulla University Hospital IRB committee.

## Abbreviations

VBAC: Vaginal Birth After Caesarean Section
TOLAC: Trial of Labor after Caesarean Section
ERC: Emergency Caesarean Section
ERCS: Emergency Repeat Caesarean Section
AUC: Area Under the Curve
ROC: Receiver Operating Characteristics.
BMI: Body Mass Index
SD: Standard Deviation
IRB: Institutional Review Board
